# ClC-5 Downregulation Induces Osteosarcoma Cell Apoptosis by Promoting Bax and tBid Complex Formation

**DOI:** 10.3389/fonc.2020.556908

**Published:** 2021-02-05

**Authors:** Fei Peng, Weisong Cai, Jianping Li, Haohuan Li

**Affiliations:** Department of Orthopedics, Renmin Hospital of Wuhan University, Wuhan, China

**Keywords:** osteosarcoma, chloride, ClC-5, apoptosis, mitochondria, Bax

## Abstract

Osteosarcoma is the most common malignant bone tumor. Chloride (Cl^−^) channels-mediated Cl^−^ movement plays an important role in regulating the functions of various cancer cells, but its role in osteosarcoma remains unclear. In this study, we found that ClC-5 was increased in osteosarcoma tissues compared with normal bone tissues. Patients with high ClC-5 expression showed poor overall survival relative to those patients with low ClC-5 expression. Higher ClC-5 expression and lower intracellular Cl^−^ concentration ([Cl^−^]_i_) were observed in osteosarcoma cells compared with normal osteoblasts. Lowering [Cl^−^]_i_ increased the viability of osteosarcoma cells, which was markedly blocked by ClC-5 downregulation. Knockdown of ClC-5 significantly induced osteosarcoma cell apoptosis and increased the release of cytochrome c from mitochondria to cytosol, concomitantly with cleavage of caspase-9, caspase-3, and PARP. The effect of ClC-5 downregulation on osteosarcoma cell apoptosis and viability was abolished by caspase-3 and caspase-9 inhibitors, but not caspase-8 inhibitor. Furthermore, ClC-5 inhibition promoted Bax translocation from cytosol to mitochondria. Immunoprecipitation showed that ClC-5 interacted with Bax and ClC-5 downregulation enhanced Bax and tBid complex formation. Collectively, we demonstrate that ClC-5 downregulation induces osteosarcoma cell apoptosis *via* mitochondria-dependent apoptotic pathway activation by promoting Bax and tBid association and subsequent Bax translocation.

## Introduction

Osteosarcoma is the most common primary malignant bone tumor in children and adolescents (1). It accounts for about 20% of primary bone cancer and 2.4% of all malignancies in pediatric patients (2). Osteosarcoma patients usually receive a three-drug chemotherapy regimen consisting of cisplatin, doxorubicin, and methotrexate, followed by surgical resection of the primary tumor in which higher survival rates have been achieved (3, 4). Unfortunately, some patients are diagnosed with advanced cancer at the first diagnosis. The therapeutic effects of surgery in this stage are poor due to the distant metastasis (1). Thus, it may be of great importance to understand the molecular mechanisms of osteosarcoma development and identify a novel biomarker that could be used for osteosarcoma diagnosis.

Chloride (Cl^−^), the most common and abundant anions in organism, plays a key role in regulating various cellular and intracellular functions (5). Cl^−^ movement resulting in decrease of intracellular Cl^−^ concentration ([Cl^-^]_i_) is closely related to diverse physiological processes, including vascular inflammation, foam cell formation, hypertension, and cancer drug resistance (6–8). It has been known that the Cl^−^ movement is strictly regulated by Cl^−^ channels, a kind of permeable channels or proteins encoded by the genes of ClC family. ClC-5, a member of ClC family, is recently suggested to be associated with the development of several kinds of cancers (9, 10). It has been reported that ClC-5 is highly expressed in glioma cells and leukemic cells (11, 12). Moreover, ClC-5 overexpression decreased drug sensitivity in multiple myeloma cells *via* promoting pro-survival autophagy (9). Nevertheless, the functional role of Cl^−^ movement and ClC-5 in osteosarcoma remains unknown. In this study, we investigated the correlation between ClC-5 and the growth of osteosarcoma cells, demonstrating that the increased ClC-5 expression may facilitate osteosarcoma cell growth and predict poor prognosis of osteosarcoma. Our findings suggest that targeting ClC-5 may be a potential strategy for the treatment of osteosarcoma.

## Methods and Materials

### Materials and Reagents

RPMI1640 medium, fetal bovine serum (FBS), penicillin, and streptomycin were obtained from Invitrogen (CA, USA). 6-methoxy-N-ethyl-1,2-dihydroquinoline (dihydro-MEQ), and antibodies against ClC-5 and GAPDH were obtained from Sigma (MO, USA). Primary antibodies against cytochrome c, cleaved caspase-9, cleaved caspase-3, cleaved caspase-8, cleaved PARP, and horse peroxidase (HRP) conjugated secondary antibodies were purchased from Cell Signaling Technology (MA, USA). Bax, tBid, and COX IV antibodies were obtained from Santa Cruz Biotechnology (CA, USA). Specific caspase-9 inhibitor Z-LEHD-FMK, specific caspase-8 inhibitor Z-IETD-FMK, and specific caspase-3 inhibitor Z-VAD-FMK were purchased from Calbiochem (Darmstadt, Germany).

### Control and Patient Osteosarcoma Samples

This study included 30 osteosarcoma patients who received surgical biopsy with no prior chemotherapy or radiation therapy between 2007 and 2008 in the Renmin Hospital of Wuhan University in Wuhan, China. Meanwhile, 30 cases of normal bone tissues were considered as control. The normal bone tissues were resected within at least 5 cm to the margin of tumor. After surgical resection, all the osteosarcoma and normal bone tissues were frozen within 30 min.

### Immunohistochemistry

Immunohistochemistry for ClC-5 was performed on 4-μm paraffin-embedded sections from human osteosarcoma tissues using the streptavidin-biotin-peroxidase complex system according to the supplier’s instructions (DAKO Japan, Tokyo, Japan). The sections were heated for 30 min at 65°C, dewaxed in xylene, and rehydrated by 100, 95, 70, and 50% alcohols at room temperature for 1 min. The sections were blocked by 10% goat serum and incubated with ClC-5 antibody overnight at 4°C, followed by incubation with biotinylated secondary antibody. The expression of ClC-5 was visualized with the streptavidin-peroxidase reaction using 3,3’-diaminobenzidine under CKX41 optical microscope (Olympus, Japan).

### Cell Culture and Transfection

The human normal osteoblasts hFOB1.19, osteosarcoma cell lines U-2OS, SaoS-2 and HOS, and human renal proximal tubule epithelial HK-2 cells were purchased from China Center Type Culture Collection (CCTCC, Shanghai, China). Cells were cultured in RPMI1640 supplemented with 10% FBS, 100 U/ml penicillin and 100 μg/ml streptomycin in a humidified atmosphere of 5% CO2 at 37°C. The sequence of the stealth siRNA duplex oligoribonucleotides against human ClC-5 gene (5’-GCACTTCCATCATTCATTT-3’) and negative siRNA were synthesized and purchased from Santa Cruz Biotechnology. The siRNAs were transfected transiently with Lipo RNAi max Reagent (Applied Biosystems, CA, USA) for 48 h according to the manufacturer’s instructions.

### Low Cl^−^ Medium Preparation

Low Cl^−^ medium was prepared as previously described (8). Briefly, RPMI1640 medium lacking NaCl and KCl was also obtained from Invitrogen. The 5 mmol/L KCl and 105 mmol/L NaCl in normal Cl^−^ medium were replaced by 5 mmol/L potassium gluconate and 105 mmol/L sodium gluconate (pH = 7.2). The osmolarities of the solutions were measured by a freezing point depression osmometer (OSMOMAT030, Gonotec GmbH, Berlin, Germany).

### Intracellular Cl^−^ Concentration ([Cl^−^]_i_) Measurement

[Cl^−^]_i_ was measured as previously described (6, 8). Osteosarcoma cells were incubated with dihydro-MEQ (100 μmol/L) in a Ringer’s buffer solution containing (in mmol/L: 119 NaCl, 2.5 KCl, 1.0 NaH_2_PO_4_, 1.3 MgSO_4_, 2.5 CaCl_2_, 26 NaHCO_3_, 11 glucose, pH 7.4) at room temperature in the dark for 30 min. Then dihydro-MEQ was oxidized to MEQ. The fluorescence of MEQ was monitored by MetaFluor Imaging software (Universal Imaging Systems, Chester, PA, USA) with 350-nm excitation and 435-nm emission wavelength. [Cl^−^]_i_ was calculated by the Stern–Volmer equation: (FO/F) – 1 = K SV [Q]. FO is the fluorescence intensity without quencher; F is the fluorescence intensity in the presence of quencher; [Q] is the concentration of quencher; and KSV is the Stern–Volmer constant.

### Cell Viability Assay

Cell viability was determined by cell counting kit-8 (CCK-8; Yiyuan Biotechnology, Guangzhou, China) according to the manufacturer’s protocols. After corresponding treatment, 10 μl of CCK8 was added to each well for additional 3 h at 37°C. The absorbance of each well was measured with a SPECTRA MAX190 spectrophotometry (Sunnyvale, CA, USA) at 450 nm wavelength.

### Real-Time PCR

The osteosarcoma samples were homogenized in TRIzol reagent (Invitrogen, CA, USA) and total RNA was isolated according to the manufacturer’s instructions. One microgram of RNA was reverse-transcribed to cDNA according to the manufacturer’s instructions (Thermo Fisher Scientific Inc., IL, USA). A SYBR QPCR Kit (Toyobo, Osaka, Japan) was used in associated with ABI 7500 real-time PCR system (Applied Biosystems, CA, USA) to detect ClC-5 mRNA expression. Human 18S rRNA was used as an endogenous control. The primer sequences were as follows: ClC-5, 5’-GTGAGGGAGAAATCCAGA-3’ and 5’-TTGATGATCAGCGTCCA-3’; 18S rRNA, 5’-CGGCTACCACATCCAAGGAA-3’ and 5’-CTGGAATTACCGCGGCT-3’.

### Western Blot Analysis and Immunoprecipitation

Osteosarcoma cells were harvested and lysed in RIPA lysis buffer containing protease and phosphatase inhibitor cocktail (Beyotime, Jiangsu, China) and the protein was isolated according to the manufacturer’s instructions. The protein content was quantified using a micro BCA kit (Beyotime). The samples containing equal protein (100 μg) were diluted in loading buffer and heated for 10 min at 99°C, before separated on 8–12% SDS-PAGE. Separated proteins were transferred onto polyvinylidene fluoride membranes (Millipore, MA, USA) at 200 mA for 90 min. Unspecific banding sites on the membrane were blocked by 5% non-fat milk. The following antibodies were used: ClC-5 (1:500), cytochrome c, cleaved caspase-9, cleaved caspase-3, cleaved caspase-8, cleaved PARP, Bax, tBid, COX IV, and GAPDH (1:1,000). After washing, membranes were incubated with appropriate HRP conjugated secondary antibody (1:1,000). Bands were detected and visualized by Amersham BiosciencesTM ECLTM Western blotting detection reagent. Densitometric analysis was conducted with Image J software (National Institutes of Health, MD, USA). For immunoprecipitation, equal cellular proteins were incubated with limiting amounts of Bax antibody overnight at 4°C with constant rotation. The complexes were collected following incubation with protein A/G agarose beads for 4 h at 4°C and resuspended in RIPA lysis buffer for western blot analysis using tBid antibody.

### Immunofluorescence Staining

To detect ClC-5 localization, osteoblast and osteosarcoma cells were fixed and labeled with ClC-5 antibody (1:100) overnight at 4°C, followed by incubation with TRITC-labeled secondary antibody (1:200, Jackson Laboratory, ME, USA) for 1 h at room temperature. Fluorescent images were acquired using the Zeiss LSM 710 laser-scanning confocal microscopy (Munich, Germany).

### Flow Cytometry

The apoptosis of osteosarcoma cells was detected with the FITC-Annexin V Apoptosis Detection Kit (Nanjing Jiancheng, Jiangsu, China) using a flow cytometry (BD Biosciences, Palo Alto, CA, USA). In brief, cells were trypsinized and harvested by centrifugation. The cell pallets were washed with PBS, re-suspended, and incubated with Annexin V and propidium iodide (PI) at room temperature in dark for 20 min. The stained cells were analyzed by a flow cytometry and data were analyzed by CFlow Plus software (BD Biosciences).

### TUNEL Staining

Cell apoptosis was also detected by TUNEL assay using an *In situ* Cell Death Detection kit (Roche, Mannheim, Germany). The cells were fixed with 4% paraformaldehyde for 30 min at room temperature, permeabilized with 0.1% Triton X-100 for 5 min, and incubated with TUNEL Reaction Mixture for 60 min at 37°C. The nuclei were counterstained with DAPI. Apoptotic cells were viewed using the Zeiss LSM 710 laser-scanning confocal microscopy. The number of TUNEL positive cells (red) were counted using Image J software. The percentage of TUNEL-positive cells was expressed as TUNEL positive cells/field.

### Mitochondrial Fractions Preparation

Mitochondrial and cytoplasmic proteins were isolated using the Mitochondria Isolation Kit (Thermo Fisher Scientific Inc., IL, USA) according to the manufacturer’s protocol. These fractions were analyzed by western blot analysis. COX IV was used as a loading control for the mitochondria fraction.

### Statistical Analyses

Continuous variables are expressed as mean ± SEM. The ANOVA (*post hoc* test: Tukey) was used to analyze the differences between groups for experiments with more than two subgroups. Survival curves were estimated using Kaplan-Meier method and compared by log rank test. Data were statistically analyzed by SPSS15.0 statistical software (SPSS Inc., IL, USA). P value <0.05 were considered statistically significant.

## Results

### Increased ClC-5 Expression in Osteosarcoma Tissues

**Figure 1:**
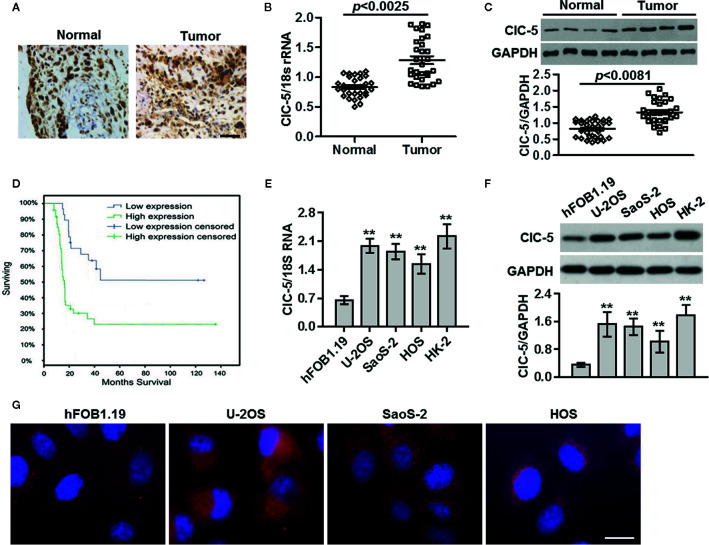
ClC-5 expression is increased in osteosarcoma tissues. **(A)** Paraffin-embedded sections of normal bone tissues and osteosarcoma tissues were collected. Expression of ClC-5 was detected by immunohistochemistry. Scale bar, 100 μm. **(B)** Real-time PCR analysis to quantify the endogenous expression of ClC-5 in normal bone tissues (n = 30) compared with osteosarcoma tissues (n = 30). **(C)** ClC-5 protein expression was determined by Western blotting and quantitative results of ClC-5 expression were presented. **(D)** Correlation of ClC-5 expression and overall survival in patients with osteosarcoma cancer. Observations were censored at trial discontinuation for reasons other than death or at trial completion. **(E, F)** The ClC-5 expression in human normal osteoblasts hFOB1.19, osteosarcoma cell lines U-2OS, SaoS-2, and HOS, and renal proximal tubule epithelial HK-2 cells was determined by real-time PCR **(E)** and western blotting **(F)**. **P < 0.01 *vs.* hFOB1.19 cells, n = 6. **(G)** Representative images of ClC-5 distributions in osteoblast and osteosarcoma cells. N = 4. Scale bar, 20 μm.

To investigate the role of ClC-5 in osteosarcoma, we first utilized immunohistochemistry to study the expression files of ClC-5 in normal bone tissues and osteosarcoma tissues. As shown in **Figure 1A**, in human osteosarcoma tissues, ClC-5 was significantly overexpressed compared with normal bone tissues. Similar to this result, both real-time PCR and western blotting revealed that the expression of ClC-5 was increased in osteosarcoma tissues compared with normal bone tissues (**Figures 1B, C**). To further determine the relationship between ClC-5 expression and the clinic pathologic characteristics of osteosarcoma patients, the patients were divided into low expression group and high expression group according to the median expression of ClC-5. We found that the ClC-5 expression was in association with tumor size, grade, distant metastasis, and clinical stage. However, the expression of ClC-5 in osteosarcoma patients had no correlation with age and gender (**Table 1**). Moreover, Kaplan-Meier survival analysis showed that ClC-5-high patients were significantly connected with poor prognosis, indicating that the osteosarcoma patients with high ClC-5 expression have lower overall survival than those with ClC-5-low expression (P = 0.02 log-rank test) (**Figure 1D**). *In vitro*, the abundance of expression of ClC-5 was significantly higher in osteosarcoma cell lines compared with normal osteoblasts hFOB1.19, and even close to that in renal proximal tubule epithelial HK-2 cells (**Figures 1E, F**), where ClC-5 has been demonstrated to be highly expressed (13). The subcellular localization of ClC-5 in osteoblast and osteosarcoma cells has not been previously explored. Immunofluorescence staining revealed that ClC-5 accumulated around the nucleus, and exhibited a punctate distribution, which extended throughout the cell (**Figure 1G**). Collectively, these results suggest that increased expression of ClC-5 may be associated with the development of osteosarcoma.

**Table 1:** Relationship between ClC-5 expression and the clinical characteristics of the osteosarcoma patients.

	ClC-5 expression		P values
	Low (n = 14)	High (n = 16)	
Age (years)			0.8731
≥18	10	11	
<18	4	5	
Gender			0.3006
Female	8	12	
Male	6	4	
Tumor size (cm)			0.0111
<10	9	3	
≥10	5	13	
Grade			0.0024
Low	8	1	
High	6	15	
Distant metastasis			0.0235
Negative	11	6	
Positive	3	10	
Stage			0.0480
I	8	3	
II	5	7	
III	1	6	

### ClC-5-Mediated Cl^−^ Efflux Promotes the Growth of Osteosarcoma Cells

**Figure 2:**
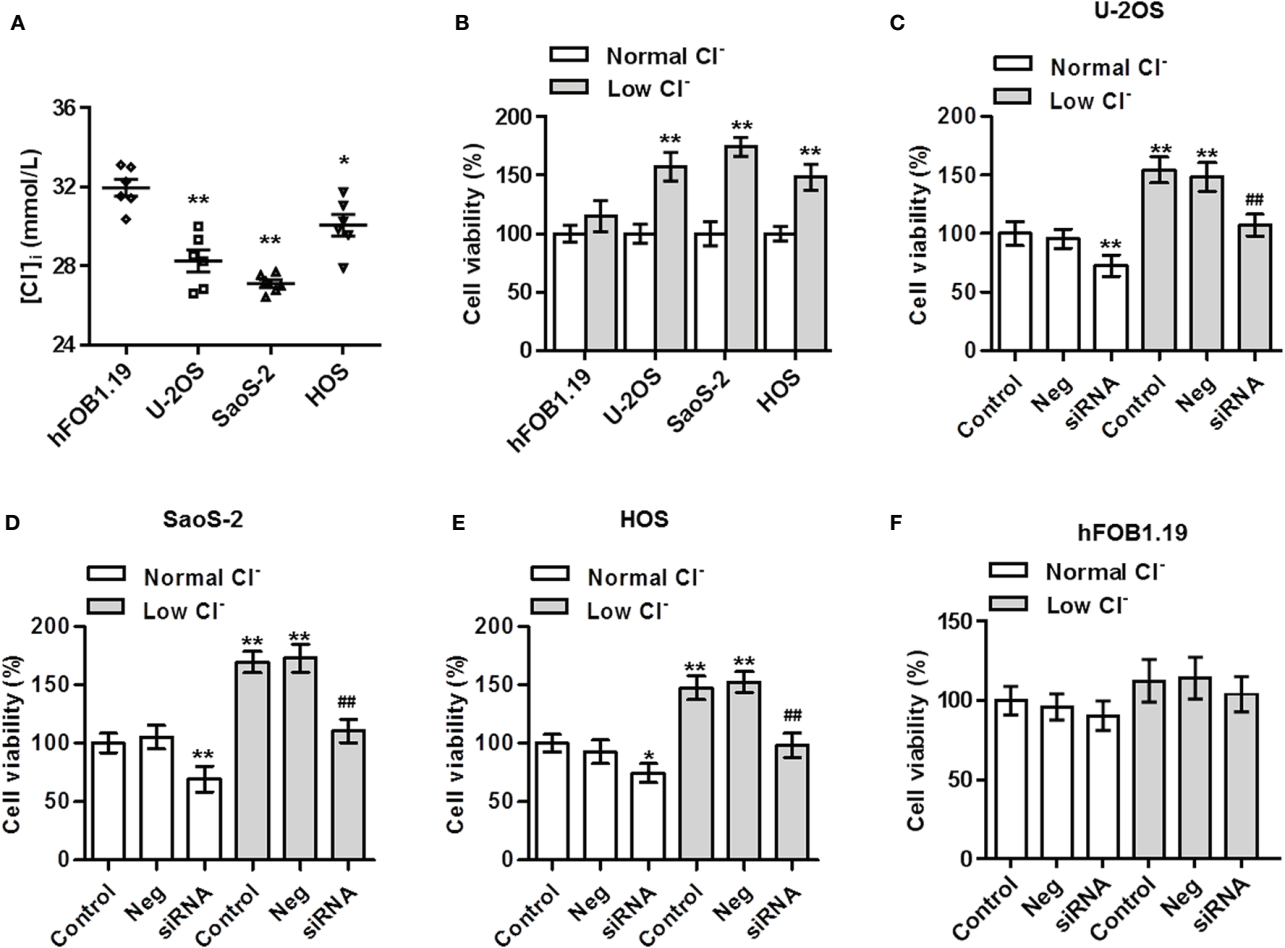
Inhibition of ClC-5 attenuates low Cl^−^-induced the increase in osteosarcoma cell viability. **(A)** The intracellular chloride concentration ([Cl^−^]_i_) in hFOB1.19, U-2OS, SaoS-2, and HOS cells was examined using MEQ fluorescence probe. *P < 0.05, **P < 0.01 *vs.* hFOB1.19 cells, n = 6. **(B)** hFOB1.19, U-2OS, SaoS-2, and HOS cells were incubated in normal medium or low Cl^−^ medium for 24 h. Cell viability was assessed by CCK-8 assay. **P < 0.01 *vs.* normal Cl^−^, respectively, n = 6. **(C–F)** U-2OS **(C)**, SaoS-2 **(D)**, HOS **(E)**, and hFOB1.19 **(F)** cells were transfected with ClC-5 siRNA or negative siRNA (Neg) for 48 h and then treated with low Cl^−^ medium for 24 h. Cell viability was determined. **P < 0.01 *vs.* normal Cl^−^+control; ^##^P < 0.01 *vs.* low Cl^−^+control, n = 6.

As ClC-5 is an important Cl^-^ channel, we thus examined whether [Cl^−^]_i_ is changed in osteosarcoma cells. The results showed that [Cl^−^]_i_ was markedly decreased in osteosarcoma cells compared with normal osteoblasts (**Figure 2A**). To explore the physiological function of the reduced [Cl^−^]_i_, low Cl^−^ medium was prepared and cell viability was assessed. CCK-8 assay revealed that lowering [Cl^−^]_i_ increased the viability of U-2OS, SaoS-2, and HOS cells, but had no effect on control osteoblasts cell line hFOB1.19 (**Figure 2B**). We next investigated that whether the increased ClC-5 expression is responsible for the lowering [Cl^−^]_i_-induced osteosarcoma cell growth, ClC-5 expression was inhibited by siRNA under normal and low Cl^−^ conditions (**Figure S1**). Inhibition of ClC-5 not only reduced osteosarcoma cell viability in normal Cl^−^ medium, but also attenuated lowering [Cl^−^]_i_-induced the increase in cell viability (**Figures 2C–E**). However, ClC-5 downregulation exerted no effect on hFOB1.19 cell viability when the cells were cultured with or without low Cl^−^ medium (**Figure 2F**).

### Downregulation of ClC-5 Induces Osteosarcoma Cell Apoptosis

**Figure 3:**
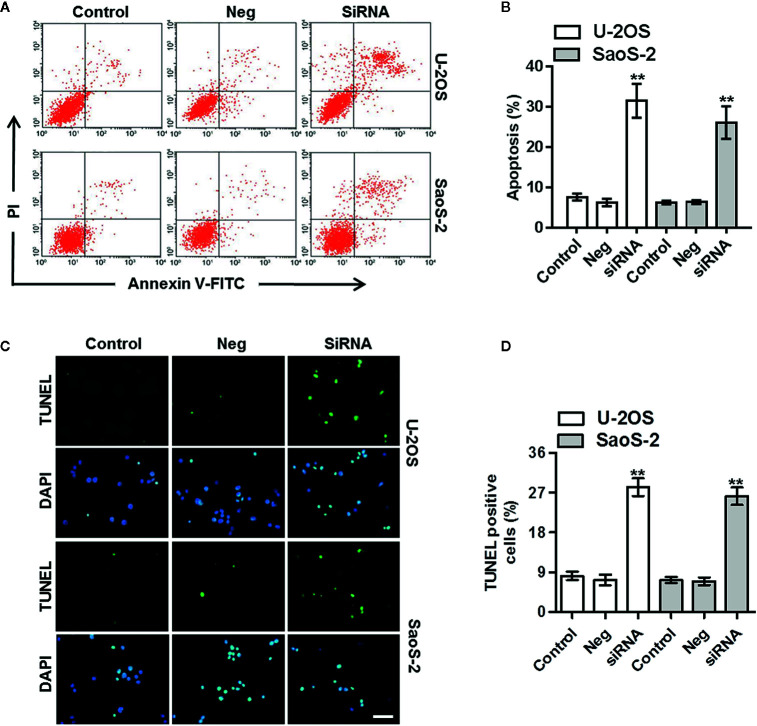
ClC-5 downregulation induces apoptosis in osteosarcoma cells. **(A)** U-2OS and SaoS-2 cells were transfected with ClC-5 siRNA or negative siRNA (Neg) for 48 h. Cell apoptosis was determined by Annexin V/PI staining. **(B)** Quantitative analysis of the percentage of apoptotic cells. **(C)** Apoptosis was also detected by TUNEL staining. Representative images were shown. **(D)** Quantification of apoptotic osteosarcoma cells. Scale bar, 20 μm. **P < 0.01 *vs.* corresponding control, n = 6.

To test whether the decreased cell viability by ClC-5 downregulation results from osteosarcoma cell apoptosis, cell apoptosis was analyzed by Annexin V-FITC/PI flow cytometry and TUNEL staining. The Annexin V-FITC/PI data showed that ClC-5 knockdown increased the apoptotic rate to 32.4 ± 5.5% and 27.8 ± 3.6% in U-2OS and SaoS-2 cells, respectively (**Figures 3A, B**). Moreover, as displayed in **Figure 3C**, there were little TUNEL-positive cells in control groups. However, the apoptotic cells were markedly increased by ClC-5 downregulation. The statistical result of the percentage of TUNEL-positive cells validated the alteration in osteosarcoma cell apoptosis (**Figure 3D**).

### ClC-5 Knockdown Enhances Osteosarcoma Cell Apoptosis *via* Mitochondria-Dependent Apoptotic Pathway

**Figure 4:**
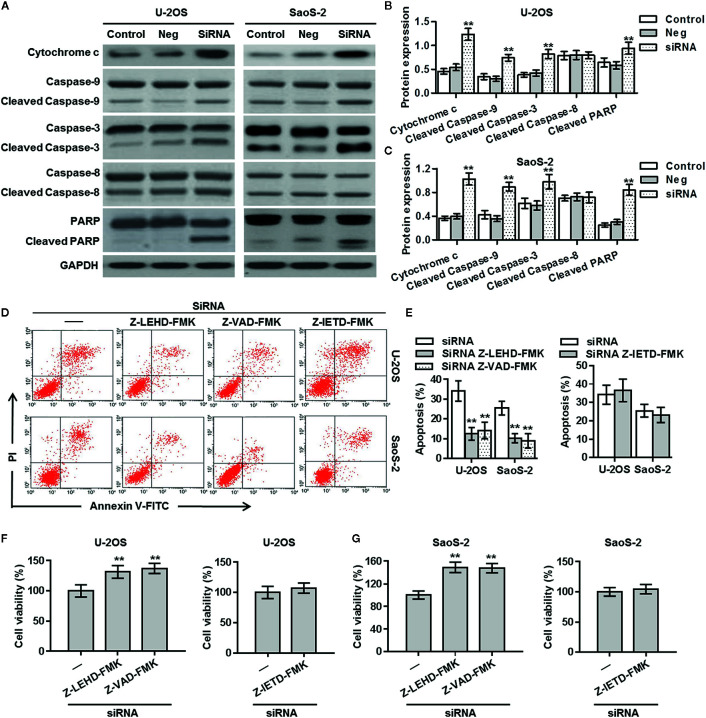
Mitochondria-dependent apoptotic pathway is involved in the effect of ClC-5 knockdown on osteosarcoma cells. **(A)** The cytosol cytochrome c level, the active forms of caspase-9/8/3, and the cleavage product of PARP were determined in U-2OS and SaoS-2 cells treated with ClC-5 siRNA or negative siRNA (Neg). **(B, C)** Densitometric analysis of apoptosis-related protein expression was performed in U-2OS **(B)** and SaoS-**2 (C)** cells. **P < 0.01 *vs.* corresponding control, n = 6. **(D, E)** U-2OS and SaoS-2 cells were pretreated with caspase-9 inhibitor Z-LEHD-FMK (10 μM), caspase-3 inhibitor Z-IETD-FMK (20 μM), or caspase-8 inhibitor Z-VAD-FMK (25 μM) for 30 min before ClC-5 siRNA transfection. Cell apoptosis was determined by Annexin V/PI staining **(D)**. Quantitative analysis of the percentage of apoptotic cells **(E)**. **P < 0.01 *vs.* siRNA, n = 4. **(F, G)** Cell viability of U-2OS **(F)** and SaoS-2 **(G)** was determined. **P < 0.01 *vs.* siRNA, n = 6.

There are two major apoptotic pathways, namely extrinsic and intrinsic (mitochondria-dependent) pathways (14, 15). To examine the mechanism how ClC-5 downregulation causes osteosarcoma cell apoptosis, we initially tested the expression of cytochrome c in the soluble cytosolic fraction. Western blotting showed that ClC-5 knockdown significantly induced cytochrome c release from mitochondria to cytosol in U-2OS and SaoS-2 cells. Furthermore, the cleavage of caspase-9/-3 and subsequent cleavage of PARP were also increased in cells transfected with ClC-5 siRNA. However, ClC-5 downregulation had no effect on the extrinsic pathway initiator caspase-8 cleavage, indicating extrinsic pathway may be not involved (**Figures 4A–C**). We next treated ClC-5 siRNA-transfected osteosarcoma cells with different caspase inhibitors and measured their effect on cell apoptosis and viability. Inhibitors experiments showed that ClC-5 downregulation-induced the increase in cell apoptosis was significantly inhibited by caspase-9 inhibitor Z-LEHD-FMK or caspase-3 inhibitor Z-VAD-FMK, but not caspase-8 inhibitor Z-IETD-FMK produced no effects (**Figures 4D, E**). Consistently, CCK-8 assay revealed that caspase-9 inhibitor and caspase-3 inhibitor but not caspase-8 inhibitor increased the viability of U-2OS and SaoS-2 cells that were transfected with ClC-5 siRNA (**Figures 4F, G**). These results indicate that intrinsic mitochondria-dependent pathway may be the major mechanism responsible for the apoptotic effects of ClC-5 downregulation in osteosarcoma cells.

### ClC-5 Interacts Bax and Induces Its Translocation

**Figure 5:**
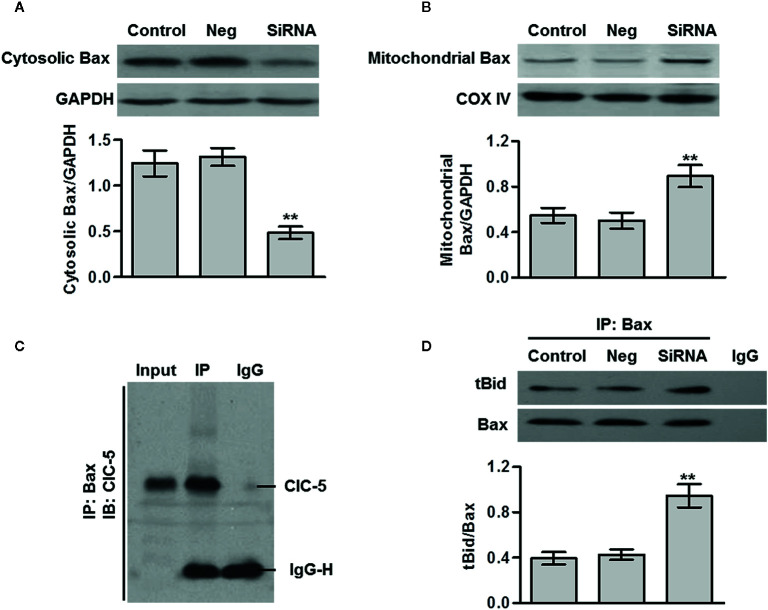
ClC-5 interacts Bax and induces its translocation. **(A and B)** U-2OS osteosarcoma cells were transfected with ClC-5 siRNA or negative siRNA (Neg) for 48 h. The Bax expression in cytosol **(A)** and mitochondria **(B)** fractions was determined by western blotting. **P < 0.01 *vs.* control, n = 6. **(C)** Cell lysates were immunoprecipitated (IP) with Bax antibody and the immunoprecipitated proteins were blotted with ClC-5 antibody. **(D)** Cell lysates from each group were immunoprecipitated with Bax antibody and blotted with tBid antibody. **P < 0.01 *vs.* control, n = 4.

The release of cytochrome c is usually triggers by the translocation of Bax from cytosol to mitochondria (15). As shown in **Figures 5A, B**, ClC-5 inhibition markedly decreased the Bax content in cytosol and increased the content in mitochondria in U-2OS cells, indicating the ClC-5 knockdown induces Bax translocation from cytosol to mitochondria. The immunoprecipitation assay in U-2OS cells showed that ClC-5 was interacted with Bax (**Figure 5C**). It has been reported that tBid directly binds with Bax and promotes Bax translocation, leading to apoptosis initiation (16, 17). We thus investigated whether ClC-5 influences the association between tBid and Bax. The results showed that ClC-5 downregulation significantly increased the binding of Bax to tBid (**Figure 5D**). Collectively, the results suggest that ClC-5 knockdown augments the interaction between Bax and tBid, resulting to Bax translocation and apoptosis in osteosarcoma cells.

## Discussion

In this study, we found overexpression of ClC-5 in osteosarcoma tissues compared to normal bone tissues. Furthermore, our results demonstrated for the first time that ClC-5-mediated Cl^−^ efflux is critical for the growth of osteosarcoma cells. Downregulation of ClC-5 induced mitochondria-dependent apoptosis in osteosarcoma cells. These data suggest that ClC-5 may play a critical role in future osteosarcoma treatment strategy.

Increasing evidence has shown the importance of Cl^−^ channels in regulating the development of different tumors. For example, ClC-3 was found to be upregulated in glioma, breast, and cervical tumors (18–20). Overexpression of ClC-3 promoted the migration and invasion of nasopharyngeal and glioma cancer cells (21, 22). Inhibition of LRRC8A could effectively attenuate cancer drug resistance in glioma and ovarian cancer cells (6, 23). Additionally, knockdown of CFTR increased the sensitivity of prostate cancer cells to cisplatin treatment (24). However, the functions of ClC-5 in cancer cells are poorly understood. ClC-5 has been found to be expressed in glioma cells and different leukemic cell lines (11, 12). ClC-5 overexpression inhibited bortezomib-induced the death of multiple myeloma cells (25). In this study, we are the first to demonstrate that ClC-5 expression was upregulated in osteosarcoma tissues. Consistent with these results, ClC-5 expression was also increased in osteosarcoma cell lines compared with normal osteoblasts. The enrichment of ClC-5 in osteosarcoma cells was even close to that in renal proximal tubule epithelial HK-2 cells, where ClC-5 has been found to be highly expressed (13). Importantly, patients with ClC-5-high expression showed poorer survival relative to ClC-5-low patients, suggesting that the increased ClC-5 expression may predict worst survival and serve as a critical prognostic indicator for osteosarcoma survival. Thus, these results indicate a key role of ClC-5 in osteosarcoma.

Interestingly, we found that the [Cl^-^]_i_ was decreased in osteosarcoma cells. Disturbance in Cl^−^ homeostasis is widely implicated in the regulation of cell proliferation and apoptosis, including cancer cells (6–8). Yang et al. observed decreased Cl^−^ efflux in temozolomide-resistant glioma cells, which was associated with inhibited cell apoptosis induced by temozolomide (6). Thus, in this study, we also explored whether the alteration in [Cl^−^]_i_ was involved in regulating the function of osteosarcoma cells. The results demonstrated that lowering [Cl^−^]_i_ using low Cl^−^ culture medium increased the viability of osteosarcoma cells. However, the increased cell viability was inhibited by ClC-5 downregulation. This strongly indicates that ClC-5-mediated Cl^−^ efflux promotes the growth of osteosarcoma cells. Subsequently, downregulation of ClC-5 markedly induced osteosarcoma cell apoptosis. It should be noted that lowering [Cl^−^]_i_ or downregulation of ClC-5 produced no significant effects on control hFOB1.19 cell viability, which may be associated with low abundance of ClC-5 in normal osteoblasts cells. These results further emphasize the importance of ClC-5 in the therapy of osteosarcoma.

It is well recognized that cell apoptosis is controlled by two major pathways: namely the extrinsic death receptor-mediate pathway and intrinsic then activated caspase-9, caspase-3, and PARP (14, 15). The former is activated at the plasma membrane *via* binding to extracellular ligand, such as FasL and TRAIL, leading to the cleavage of caspase-8 (26, 27). On the other hand, the activation of intrinsic pathway results in cytochrome c release from mitochondria to cytosol, which in turn cleaves caspase-9, caspase-3, and PARP (14). Thus, each apoptotic pathway activates corresponding initiator caspase, caspase-8, or caspase-9. In this study, we found that ClC-5 inhibition increased the release of cytochrome c followed by activation of caspase-9, caspase-3, and PARP. However, ClC-5 knockdown had no effects on caspase-8 activation, suggesting that ClC-5 downregulation activates the intrinsic pathway rather than extrinsic pathway. This was further supported by the subsequent results that caspase-9 inhibitor or caspase-3 inhibitor reversed the pro-apoptotic effects of ClC-5 downregulation.

We next investigated the mechanisms how ClC-5 knockdown causes cytochrome c release and initiates apoptosis. It is worthy to note that the release of cytochrome c is triggered by the activation of Bax (16). Under normal conditions, Bax is mainly expressed in cytosol. Upon pro-apoptotic stimulation, Bax translates from cytosol to mitochondria and increases the membrane permeability, leading to cytochrome c release and apoptosis initiation (28, 29). We found that ClC-5 downregulation induced Bax translocation from cytosol to mitochondria. More importantly, we reported for the first time that ClC-5 is a novel Bax-interacting protein. Given that BH3-only protein, such as tBid, can bind with Bax and help the translocation of Bax from cytosol to mitochondria (17, 30), the effects of ClC-5 knockdown on the interaction between Bax and tBid. We observed that ClC-5 downregulation increased the binding of Bax to tBid, suggesting that ClC-5 and tBid may competitively bind with Bax.

In conclusion, our results demonstrate that ClC-5 downregulation induces osteosarcoma cell apoptosis, which involves promotion of Bax and tBid complex formation, leading to cytochrome c release and intrinsic mitochondria-dependent apoptotic pathway activation. These findings suggest that ClC-5 may be a promising target for the treatment of osteosarcoma.

## Data Availability Statement

The raw data supporting the conclusions of this article will be made available by the authors, without undue reservation.

## Ethics Statement

The studies involving human participants were reviewed and approved by Renmin Hospital of Wuhan University. The patients/participants provided their written informed consent to participate in this study. Written informed consent was obtained from the individual(s) for the publication of any potentially identifiable images or data included in this article.

## Author Contributions

Conception and design: FP. Administrative support: FP. Provision of study materials or patients: WC and JL. Collection and assembly of data: FP and HL. Data analysis and interpretation: FP. Manuscript writing: all authors. All authors contributed to the article and approved the submitted version.

## Funding

This work was supported by the National Natural Science Foundation of China (631308110).

## Conflict of Interest

The authors declare that the research was conducted in the absence of any commercial or financial relationships that could be construed as a potential conflict of interest.
