# Supplementary material for: ClC-5 Downregulation Induces Osteosarcoma Cell Apoptosis by Promoting Bax and tBid Complex Formation
Source: Front Oncol. 2021 Feb 5;10:556908. doi: 10.3389/fonc.2020.556908 (PMC7892965; doi:10.3389/fonc.2020.556908)
Supplement: Supplementary file 1 [file DataSheet_1.docx]

ClC-5 downregulation induces osteosarcoma cell apoptosis by promoting Bax and tBid complex formation

Fei Peng^1*^, Weisong Cai^1^, Jianping Li^1^, Haohuan Li^1^

^1^ Department of Orthopedics, Renmin Hospital of Wuhan University, Wuhan, Hubei, 430060, China.

*** Correspondence:**Fei Peng
pengf_whu@sina.com

**Figure S1**

**
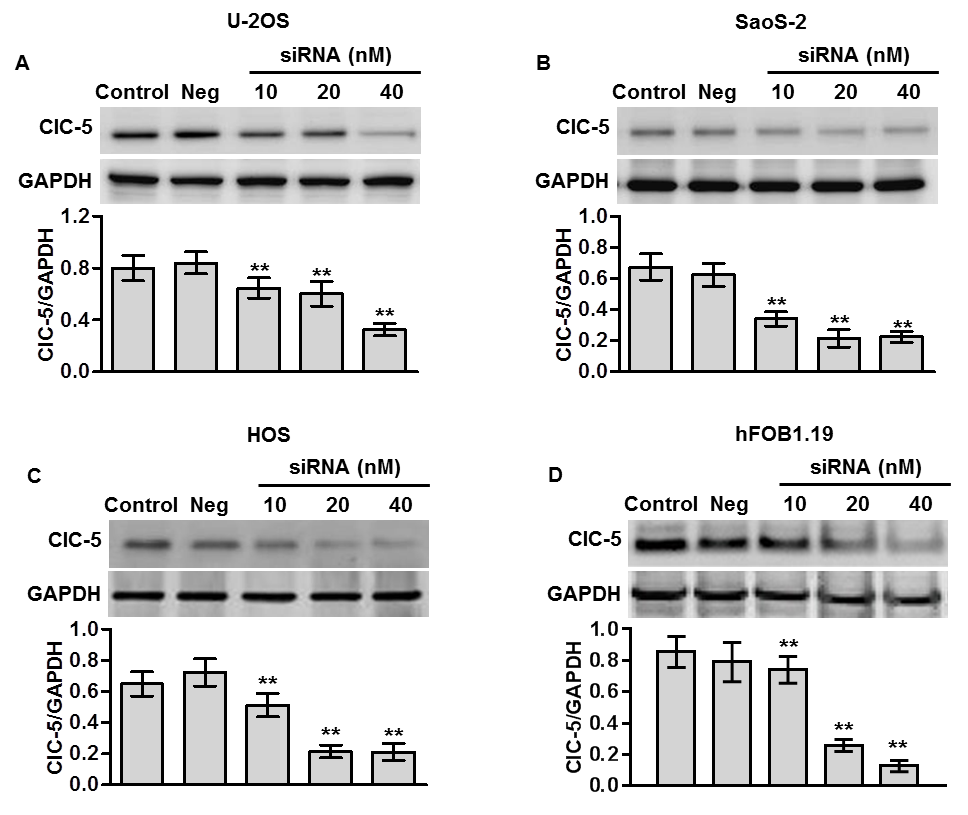
**

**Figure S1. Effects of ClC-5 siRNA on ClC-5 expression in osteosarcoma and osteoblasts cells.** (A-D) U-2OS (A), SaoS-2 (B), HOS (C) and hFOB1.19 (D) cells were transfected with negative siRNA (Neg), or different concentrations of ClC-5-targeting siRNA (siRNA) (10, 20 and 40 nM) for 48 h. ClC-5 expression was determined by western blotting. **P<0.01 vs. control, n=4.
